# Quick and simple; psoas density measurement is an independent predictor of anastomotic leak and other complications after colorectal resection

**DOI:** 10.1007/s10151-019-1928-0

**Published:** 2019-02-21

**Authors:** P. J. J. Herrod, H. Boyd-Carson, B. Doleman, J. Trotter, S. Schlichtemeier, G. Sathanapally, J. Somerville, J. P. Williams, J. N. Lund

**Affiliations:** 1 Division of Medical Sciences and Graduate Entry Medicine, Department of Surgery, University of Nottingham, Derby, UK; 2 Medical Research Council-Arthritis Research UK Centre for Musculoskeletal Ageing Research, Royal Derby Hospital, University of Nottingham, Derby, DE22 3DT UK; 3 Combined Gastroenterology Research Unit, Scarborough General Hospital, Scarborough, UK

**Keywords:** Sarcopenia, Colorectal neoplasms, Postoperative complications, Anastomotic leak

## Abstract

**Background:**

Radiologically defined sarcopenia has been shown to predict negative outcomes after cancer surgery, however radiological assessment of sarcopenia often requires additional software and standardisation against anthropomorphic data. Measuring psoas density using hospital Picture Archiving and Communication Systems (PACS), universally available in the UK, may have advantages over methods requiring the use of additional specialist and often costly software. The aim of this study was to assess the association between radiologically defined sarcopenia measured by psoas density and postoperative outcome in patients having a colorectal cancer resection.

**Methods:**

All patients having a resection for colorectal cancer, discussed by the colorectal multi-disciplinary team in one institution between 1/1/15 and 31/12/15, were retrospectively identified. Mean psoas density at the level of the L3 vertebra was analysed from preoperative computed tomography (CT) scans to define sarcopenia using the Picture Archiving and Communication Systems (PACS). Postoperative complications and mortality were recorded.

**Results:**

One hundred and sixty-nine patients had a colorectal resection for cancer and 140 of these had a primary anastomosis. Ninety-day mortality and 1-year mortality were 1.1% and 7.1%, respectively. Eighteen (10.7%) patients suffered a Clavien–Dindo grade 3 or 4 complication of which 6 (33%) were anastomotic leaks. In the whole cohort, sarcopenia was associated with an increased risk of Clavien–Dindo grade 3 or 4 complications [adjusted OR 6.33 (1.65–24.23) *p* = 0.007]. In those who had an anastomosis, sarcopenia was associated with an increased risk of anastomotic leak [adjusted OR 14.37 (1.37–150.04) *p* = 0.026].

**Conclusions:**

A quick and easy radiological assessment of sarcopenia by measuring psoas density on preoperative CT scan using software universally available in the UK is highly predictive of postoperative morbidity in colorectal cancer patients.

## Introduction

Sarcopenia, the age-related loss of muscle or lean mass is a marker of frailty which is associated with increased postoperative morbidity and mortality and can be identified at preoperative cross-sectional imaging [1–3]. Measurement of muscle area at the third lumbar (L3) vertebral level on CT scan, suggests sarcopenia is associated with increased postoperative complications, 30-day and 90-day mortality, and reduced 1, 3 and 5 year survival [1]. Poor outcomes have also been associated with sarcopenia in surgery for gastrointestinal cancer [4, 5].

Incorporating a radiological assessment of sarcopenia into the preoperative assessment of potentially may identify patients most at risk of postoperative complications, individualising risk for improved preoperative counselling, identifying those who may benefit from prehabilitation interventions and perhaps influencing operative strategy such as whether to perform a primary anastomosis without a stoma [6, 7].

To date, most series have used either psoas area or other abdominal muscle cross-sectional areas to radiologically define sarcopenia and its effect on outcomes. However, these methods require standardisation for height, weight and sex and quantification of cross-sectional area needs to be performed on software packages separate from those used for the main viewing and clinical reporting of images, making these methods difficult to generalise [1].

Calculation of psoas density may be a more accurate method of determining sarcopenia than psoas area, as variable fat content of the skeletal muscle may increase cross-sectional area confounding the measurement of lean muscle area and does not require standardisation for height and weight [8]. Psoas density better predicted poor outcomes compared to cross-sectional area in a cohort of trauma patients [9], a cohort having pancreatectomy [10], a cohort of patients having cardiac surgery [11] and patients having an emergency laparotomy [12]. However, most studies used software separate to the main imaging viewing and reporting software.

Measurement of psoas density can be performed quickly and easily with good reliability after minimal training, using the Picture Archiving and Communication Systems (PACS) available to all hospital trusts in the United Kingdom and therefore can be done at the time of reporting of the images if required, or can be calculated by any user of the standard PACS system, accessible to all clinicians [12]. If measurement of psoas density is useful for risk prediction then it could more easily be generalised using this method.

The aim of this study was to evaluate the association between radiologically defined sarcopenia (by psoas density using standard hospital PACS image viewing software) and postoperative complications and other outcomes for patients having bowel resection for the treatment of colorectal cancer.

## Materials and methods

This retrospective observational study was reported in accordance with the Strengthening the Reporting of Observational studies in Epidemiology (STROBE) statement [13].

### Case identification

All patients discussed at the Colorectal Cancer Multidisciplinary team meeting in one institution in 1 calendar year (1/1/2015–31/12/2015) were identified from hospital cancer department records. This was cross-referenced with operating theatre records to identify patients having a major colorectal resection for colorectal cancer with initial curative intent.

### Radiological data

**Fig. 1:**
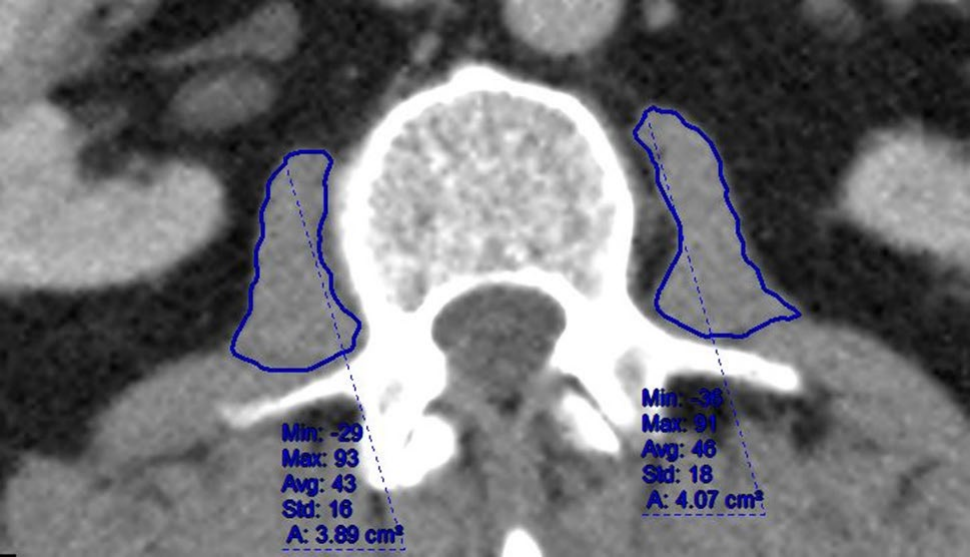
Measurement of psoas density

Preoperative staging CT scan images were obtained for all patients and psoas density data extracted by one trained operator (SS), blinded to the outcome of the patient as using the method previously described [12]. In brief, using the hospital standard PACS imaging software (Centricity Universal Viewer Version 6.0, GE Healthcare, Chicago, USA), freehand regions of interest were drawn around both psoas muscles in one CT slice at the L3 vertebrae level where both transverse processes were visible (Fig. 1). The PACS calculated density in Houndsfield units (HU) recorded. This was performed for both psoas and the arithmetic mean density calculated. A random sample of 5 cases was measured on a second occasion observed and timed by a second independent trained author (PH) to ensure standardisation.

### Outcome data

Preoperative patient details and postoperative outcome data were retrieved from electronic patient records. Returns to theatre were cross-referenced with operating theatre records to ensure complete data capture. Complications were classified according to the Clavien–Dindo (CD) system with CD3/4 complications defined as significant [14].

### Statistical analysis

Descriptive data are presented as mean (± standard deviation), median (IQR) or number (%) as appropriate. To test the association between mean psoas density and CD3/4 complications, leak and mortality we performed logistic regression. We adjusted for the following confounders as defined by the previous literature; age, gender, cardiovascular c-morbidity, preoperative renal impairment, presence of anaemia, low albumin, Body Mass Index category and whether the patient had received neoadjuvant long course chemoradiotherapy [15–18]. Sex, cardiovascular co-morbidity, renal impairment, anaemia and, albumin and neoadjuvant of long course chemo-radiotherapy were modelled as binary data. For BMI, we created dummy variables for each Body Mass Index (BMI) category using the i.prefix in STATA [STATA Version 15 (StataCorp, College Station, TX, USA)]. Complete data sets of confounders and patient outcome were available for statistical analysis.

We assessed linearity of predictors using the lowess command in STATA with logit transformed outcomes. For logistic models we assessed calibration using calibration plots and goodness of fit using the Hosmer–Lemeshow goodness of fit test. Due to non-linearity with haemoglobin and albumin these were modified as binary for patients who were anaemic and had low albumin, respectively. Due to issues with separation in the anastomotic leak models we used Firth logistic models with penalised maximum likelihood estimates.

Initially we investigated the effect of mean density of the psoas muscle at L3 vertebral level on CD3/4 complications and anastomotic leak. Mean density was modelled as a linear variable. Following this, receiver operating characteristic (ROC) curves were plotted for mean density of psoas muscle against both incidence of CD3/4 complications and anastomotic leak independently. Optimal cutoffs to define sarcopenia were calculated using these curves. We present separate analyses for the effect of mean density of the psoas muscle on both outcomes and also the effect of sarcopenia (binary) as defined by ROC curve for both outcomes. A further binary logistic regression was performed to investigate the impact of sarcopenia on 1-year mortality.

Intrarater reliability was assessed using a two-way mixed effects model and intraclass correlation coefficients (ICC) reported with a 95% CI [19].

Odds Ratios (OR) are presented with 95% confidence intervals (CI) and p values. Significance was taken at the level of *p* ≤ 0.05. We conducted all analyses using STATA Version 15 (StataCorp, College Station, TX USA.)

## Results

During the study period 169 patients had a colorectal resection for treatment of colorectal cancer with curative intent. One hundred forty patients had a primary gastrointestinal anastomosis. Patient demographics are shown in Table 1 and operations performed in Table 2.

**Table 1:** Patient demographics

	Sarcopenic *n* (%) 51 (30)	Non sarcopenic *n* (%) 118 (70)	Total *n* (%) 169 (100)
Mean (SD) age (years)	72 (10)	66 (11)	68 (11)
Male *n* (%)	34 (67)	56 (47)	91 (54)
Median (IQR) length of stay (days)	8 (6–13)	6 (4–11)	7 (4–11)
Mean (SD) psoas density	38.2 (5.1)	53.0 (5.7)	49 (9)
Primary anastomosis *n*(%)	42 (82)	98 (83)	140 (83)
Clavien–Dindo 3/4 complication *n* (%)	10 (19)	8 (7)	18 (11)
Anastomotic leak *n* (%)	4 (8)	2 (2)	6 (4)
Emergency presentation *n* (%)	13 (25)	9 (8)	22 (13)

**Table 2:** Operations performed

Operation	*N* (%)
Anterior resection	76 (45)
Right hemicolectomy	54 (32)
Abdominoperineal excision of rectum	15 (9)
Extended right hemi colectomy	12 (7)
Subtotal/panproctocolectomy	7 (4)
Hartman’s resection	5 (3)

Overall 90-day mortality and 1-year mortality were 1.1% and 7.1%, respectively. Median (IQR) psoas density for the whole cohort was 48.5 (43–54.5) HU. In total, 18 (10.7%) patients suffered a CD3/4 complications of which 6 were confirmed anastomotic leaks requiring reoperation.

### Effect of psoas mean density

For the group as a whole, increasing mean psoas density was associated with a decreased risk of CD3/4 complication [unadjusted OR 0.92 (0.87–0.98) *p* = 0.007].This risk remained after adjustment for the other confounding variables [adjusted OR 0.89 (0.83–0.96) *p* = 0.003].

In those patients having an anastomosis, increasing psoas density was associated with a decreased risk of an anastomotic leak [unadjusted OR 0.90 (0.82–0.99) *p* = 0.039]. This risk remained after adjustment [adjusted OR 0.88 (0.8–0.98) *p* = 0.019].

### Setting optimal cutoffs of mean psoas density to predict outcomes (Defining radiological sarcopenia)

ROC curves were produced for mean density against both CD3/4 complications and anastomotic leaks using STATA. Optimal cutoffs to detect either endpoint were then defined using the Roctab command in STATA. These cutoffs were taken as the point giving a minimum of 75% specificity to make them clinically relevant as a rule-in test. In the whole cohort a mean density of less than or equal to 44.5 HU (specificity 75% and sensitivity 44%) was taken as the optimal cutoff. When looking at the anastomosis-only group, a mean density of less than or equal to 43.5 HU was the optimal cutoff (specificity 75% and sensitivity 33%). These cutoffs were taken as radiologically defined sarcopenia.

### Effect of sarcopenia

In the whole cohort, radiologically defined sarcopenia was associated with an increased risk of CD3/4 complication [unadjusted OR 3.35 (1.2–9.08), *p* = 0.017]. This risk was strengthened after adjustment [adjusted OR 6.33(1.6–24.24) *p* = 0.007] (Table 3).

**Table 3:** Effect of Sarcopenia on rate of postoperative complications and mortality in all colorectal resections (*N* = 169)

Outcome	Unadjusted odds ratio (95% CI)	*p* value	Adjusted odds ratio (95% CI)	*p* value
Clavien–Dindo 3/4 complication	3.35 (1.23–9.10)	0.017	6.33 (1.65–24.23)	0.007
1-year mortality	2.08 (0.62–6.95)	0.23	1.73 (0.47–6.3)	0.406

In those having an anastomosis, radiologically defined sarcopenia was associated with an increased risk of anastomotic leak [unadjusted OR 5.66 (1.00–32.2) *p* = 0.05]. This association was strengthened after adjustment for confounders [adjusted OR 14.37 (1.3–150.0) *p* = 0.026] (Table 4).

**Table 4:** Effect of sarcopenia on rate of postoperative complications and anastomotic leaks in patients having a primary anastomosis (*N* = 140)

Outcome	Unadjusted odds ratio (95% CL)	*p* value	Adjusted odds ratio (95% CL)	*p* value
Anastomotic leak	5.66 (1.0–32.2)	0.05	14.37 (1.37–150.0)	0.026

In the whole cohort, sarcopenia was not significantly associated with 1-year mortality [unadjusted OR 2.08 (0.62–6.95) *p* = 0.23]. This risk remained non-significant after adjustment [adjusted OR 1.7 (0.47–6.3) *p* = 0.41]. No analysis was performed for 90-day mortality due to the low event rate (only 2 deaths within 90 days).

### Standardisation

The mean (SD) time to perform measurement of density for both psoas muscles on aCT scan using PACS was 48 (7) s. The ICC for psoas evaluation was 0.95 (0.59–0.99).

## Discussion

This study has shown that sarcopenia, quantified by a simple measurement of mean psoas density on a preoperative CT scan of the abdomen, calculated quickly and easily using software available to all clinicians in the UK, is an independent predictor of significant postoperative complication or anastomotic leakage following a colorectal cancer resection. Measurement of psoas density in this fashion can be completed in less than 1 min by a trained operator and does not require image transfer to additional software, as has been the case in other series [4, 20–23]. Psoas density also has the advantage of not requiring standardisation for patient height, weight or sex, avoiding the need for any additional calculations before producing a result for comparison with the cohort. As this method can be used quickly and easily and does not need additional patient measurements or a separate software package there are significant advantages over other described techniques for calculation of a measure sarcopenia which could make this technique easy to generalise.

Ease of use of this psoas density technique may aid decision-making and recommendation in real-time in the colorectal cancer multi-disciplinary meeting and add to personalised counselling of individual patients’ own risk of complications and death, alongside existing tools. Better prediction of anastomotic leak will tailor the decision to defunction. It has always been important to counsel patients according to their individual circumstances but it is a requirement to do so after the Montgomery ruling [24]. The ability to better predict the complications for individuals will provide data to support this process. As sarcopenia has been consistently associated with postoperative complications following surgery for a wide range of gastrointestinal cancers [5], it is likely that this psoas density technique may have the potential to predict complications following surgery for other cancer types in addition to colorectal cancer.

Although sarcopenia, as defined in this study, was associated with significant complications and anastomotic leakage, we were unable to show any significant impact on mortality. However, mortality was a rare event in this series and the lack of difference may well represent a type 2 error.

One strength of this study was its use of an ROC curve analysis to define optimum cutoffs for sarcopenia in our cohort as there are no reference standards for psoas density for patients having colorectal cancer resection. Previous studies have used less statistically sound methods of determining sarcopenia, such as the use of the lower quartile of mean density [12, 23]. If this had been done in our cohort, the accuracy of our model to predict significant complications would have been reduced. Further validation studies across larger cohorts will be required to set a reference standard for a sarcopenic psoas density for patients having resection with curative intent for colorectal cancer. Of note, the lower quartile of psoas density in our cohort was significantly higher than others using this method [12] and our cutoff defined by the ROC curve was still higher. This may be due to intrinsic differences between our cohort and those that have previously been studied and requires further work to understand the underlying reasons.

The precision of our study was in part limited by the low event rate of anastomotic leak which can be addressed in a larger, multicentre validation study of our proof of concept.

### Conclusions

A quick and easy measurement of psoas density to define sarcopenia on a preoperative CT scan at the L3 vertebral level, using software universally available in the UK, can be used to predict patients most at risk of postoperative complications and anastomotic leak after colorectal cancer resection.
